# Dynamics of Soil Microbial Communities During Diazepam and Oxazepam Biodegradation in Soil Flooded by Water From a WWTP

**DOI:** 10.3389/fmicb.2021.742000

**Published:** 2021-11-29

**Authors:** Marc Crampon, Coralie Soulier, Pauline Sidoli, Jennifer Hellal, Catherine Joulian, Mickaël Charron, Quentin Guillemoto, Géraldine Picot-Colbeaux, Marie Pettenati

**Affiliations:** ^1^Bureau de Recherches Géologiques et Minières, Orléans, France; ^2^UMR 7619 METIS, Sorbonne Université, Paris, France

**Keywords:** benzodiazepines, soil, microbial community, biodegradation, WWTP

## Abstract

The demand for energy and chemicals is constantly growing, leading to an increase of the amounts of contaminants discharged to the environment. Among these, pharmaceutical molecules are frequently found in treated wastewater that is discharged into superficial waters. Indeed, wastewater treatment plants (WWTPs) are designed to remove organic pollution from urban effluents but are not specific, especially toward contaminants of emerging concern (CECs), which finally reach the natural environment. In this context, it is important to study the fate of micropollutants, especially in a soil aquifer treatment (SAT) context for water from WWTPs, and for the most persistent molecules such as benzodiazepines. In the present study, soils sampled in a reed bed frequently flooded by water from a WWTP were spiked with diazepam and oxazepam in microcosms, and their concentrations were monitored for 97 days. It appeared that the two molecules were completely degraded after 15 days of incubation. Samples were collected during the experiment in order to follow the dynamics of the microbial communities, based on 16S rRNA gene sequencing for Archaea and Bacteria, and ITS2 gene for Fungi. The evolution of diversity and of specific operating taxonomic units (OTUs) highlighted an impact of the addition of benzodiazepines, a rapid resilience of the fungal community and an evolution of the bacterial community. It appeared that OTUs from the *Brevibacillus* genus were more abundant at the beginning of the biodegradation process, for diazepam and oxazepam conditions. Additionally, Tax4Fun tool was applied to 16S rRNA gene sequencing data to infer on the evolution of specific metabolic functions during biodegradation. It finally appeared that the microbial community in soils frequently exposed to water from WWTP, potentially containing CECs such as diazepam and oxazepam, may be adapted to the degradation of persistent contaminants.

## Introduction

Due to the non-specificity of wastewater treatment plants (WWTPs) toward many micropolluants and in particular contaminants of emerging concern (CECs), these are frequently discharged to the environment. Several solutions exist to treat CECs, but significant knowledge gaps remain (Basheer, 2018). Recently, in order to minimize wastewater treatment costs regarding energy and chemicals consumption, multi-barrier treatment systems that can combine engineered systems and nature-based solution (NBS), such as soil aquifer treatment (SAT) systems, were described. Hermes et al. (2019) studied the removal processes involved in a SAT context, by focusing on trace organic chemicals and their metabolites. They found that the fate of these compounds was mainly controlled by sorption onto soil constituents (especially linked to organic matter content and clays for cationic compounds), dilution, and to the nature of the compounds themselves (cationic, non-ionic, molecular weight, etc.). Biodegradation was also described as a removal process for a variety of organic contaminants, depending especially on the specificity of microorganisms present in the system (Rauch-Williams et al., 2010; Falås et al., 2016; Regnery et al., 2016), as well as the biodegradability of the considered compounds. Natural attenuation linked to microbial communities’ adaptation represents a NBS for the remediation of CECs. The identification of biodegradation mechanisms could help to increase the efficiency of treatments in WWTPs and consequently limit environmental impacts. In this context, it is necessary to understand the adaptation capacity of endemic microbial communities exposed to CECs stress, and their possible role in CECs remediation in the case of soil and aquifer treatments.

Benzodiazepines are pharmaceutical CECs that are frequently found in water from WWTPs. They belong to a class of psychoactive drugs that contain a benzene and a diazepine ring in their core chemical structure. They have anticonvulsant, sedative, muscle relaxant, hypnotic, and anxiolytic properties. These molecules, especially diazepam (DZ), are described as poorly biodegradable when introduced into the environment (Redshaw et al., 2008). Some degradation pathways have been highlighted, such as demethylation, especially due to fungal enzymes (Helbling et al., 2010), but an efficient biodegradation in the environment has not yet been reported. Some researchers found that DZ was not directly degradable by bacteria, especially when other carbon sources that are more easily accessible are available, which is the case in soils (Tappin et al., 2014). They also showed that DZ degradation products from photodegradation are much more easily biodegraded by bacteria. The first stages of biodegradation could be linked to soil fungal activity, the bacteria then taking over the less complex metabolites. Oxazepam (OXA) seems to be more easily biodegradable by bacteria derived from WWTPs (Redshaw et al., 2008), but these benzodiazepines are generally described as poorly biodegradable (Tappin et al., 2014). DZ (Valium^®^) is certainly the best known molecule of this group and has a long-term impact because of its persistent metabolites. In humans, it is metabolized to *N*-desmethyldiazepam (also called nordazepam) (*t*_1/2_ ∼ 100 h). Nordazepam (ND, Calmday^®^) is then 3-hydroxylated to OXA (Serax^®^). The presence of the hydroxyl group allows its glucuronation and excretion in urine and explains its low half-life (∼4–15 h) compared to DZ and ND (Kosjek et al., 2012).

Benzodiazepines are halogenated molecules, which are often considered as a factor that negatively affects biodegradation. The dehalogenation of molecules with more than two to three halogen groups is often considered possible only under anaerobic conditions. Most of the data on the efficiency of wastewater treatments concern DZ and show that (i) less than 10% is removed during conventional biological treatment, (ii) the efficiency seems to be higher during anaerobic treatment (10–50%), and (iii) the removal rate is mainly related to sorption rather than degradation (Ternes et al., 2004; Löffler et al., 2005; Ghattas et al., 2017). Similarly, OXA is persistent in both aerobic and anaerobic treatment in the environment (Patterson et al., 2010). DZ appears to be subject to photodegradation in the environment, which may be a pathway for the removal of DZ from surface water (Boreen et al., 2003).

Although the Kow of benzodiazepines [DZ: 2.8, ND: 2.9, OXA: 2.2, Episuite (EPA)] does not appear to indicate that sorption plays a significant role in their removal (or delay), Ternes et al. (2004) classified DZ as readily adsorbable on activated carbon (99% removal on 0.2 mg L^–1^ activated carbon), and Calisto and Esteves (2009) showed that temazepam and OXA can undergo sorption on humic substances. On the other hand, sorption could limit bioaccessibility and thus decrease potential biodegradation. Löffler et al. (2005) showed that DZ was highly persistent with rapid and extensive sorption on sediments, stable in soils, groundwater, and during wastewater treatment. The same study showed that OXA is moderately persistent in the environment with a lower DT50 and a lower sorption rate on sediments than DZ. In addition, Löffler et al. (2005) did not observe any degradation of DZ to OXA in sediments, suggesting that it is difficult to compare the fate of DZ in the environment and in humans.

Consequently, the use of benzodiazepines to simultaneously study the processes of sorption and biodegradation—in order to quantitatively differentiate these two processes in the dissipation of molecules in soils—is needed to better understand its behavior in contaminated environments. Considered as persistent, resistant to biodegradation, and with supposed limited sorption (Kow < 3), we aimed at studying the fate of DZ and OXA in soils flooded by water from WWTP with known benzodiazepine inputs. The present study focuses on biodegradation. The objectives were to assess the capacity of soil microbial communities (bacteria, archaea, and fungi), to resist and potentially degrade benzodiazepine molecules, especially DZ and OXA. A focus was made on (i) the biodegradation of mother molecules DZ and OXA in soils, and (ii) the dynamics of bacteria, archaea, and fungi communities during the degradation kinetics. The evolution of metabolic pathways was also explored, as well as links between degradation and biodiversity.

## Materials and Methods

### Context and Soil Sampling

**FIGURE 1:**
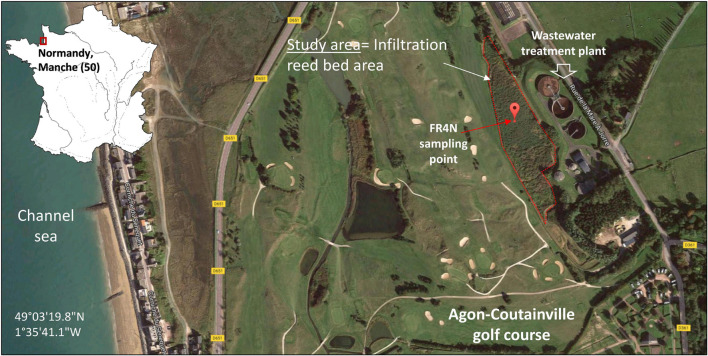
Location of the study area and sampling point FR4N in Manche department (50), Normandy, France (image modified from Google earth).

Soils were sampled in the reed-bed area of a SAT system implanted in the city of Agon-Coutainville (France, Normandy), located along the English Channel Coastline (Figure 1). The commune, with an estimated population of 2,800 residents (INSEE, 2015), is one of the oldest seaside resorts of the Manche department and harbors the largest production of shellfish in France (production and storage, ∼400 ha of shellfish parks). The conservation of the coastal ecosystem and the associated sanitary stakes are essential for local economy and have to be fully integrated to the ongoing development of residential areas, tourism, and shellfish aquaculture (approximately 7 km of coast). Subject to a large tidal range, groundwater resources are prone to salinization in this coastal area, limiting the fresh water supply. Sustainable water management in this area must consider seasonal variations of the population (during summer vacations) and the irrigation needs of a backshore golf course. In addition, environmental regulations as well as the water framework directive must be followed (DIRECTIVE 2006/7/CE), in spite of financial constraints.

To face this demanding challenge and to preserve the estuary ecosystem from secondary treated wastewater discharged to surface water (river or sea) in Agon-Coutainville, the SAT system has been considered for more than 14 years as locally more efficient and sustainable (Picot-Colbeaux et al., 2020). The SAT consists of a fed reed-bed area of 35,000 m^2^ with secondary treated wastewater provided by a biological WWTP. This area is divided into three parts alternatively flooded by the treated water with an average daily volume of 1,390 m^3^ (mean 2,005–2,019) which infiltrates into a 7- to 9-m-thick sandy aquifer. Monitoring boreholes intercept sands (fine to coarse) and silty sands, which become rich in fragments of shells with depth, and then reach the altered clay from the bed rock (schist). The first meters of the aquifer always contain organic matter.

The quality of discharged treated wastewater and downstream groundwater is monitored and respects the according French regulations although many questions remain about the fate of CECs in this system. The efficiency of this combined engineered and natural system were addressed in the European AquaNES project^1^ (Pettenati et al., 2019a,b; Picot-Colbeaux et al., 2020). Previous chemical monitoring of the study site and released water have shown non-negligible concentrations of benzodiazepines in the water at the outlet of the WWTP, especially for OXA (approximately 1,678 ng L^–1^), which is maybe also present as a DZ degradation by-product.

Five FR4N soil samples were taken from a 25 m^2^ square (four points and one in the middle) from the 0–20 cm layer and were mixed and homogenized on site, representing about 25 kg of soil. Plants residues and roots were discarded. The soil sample was analyzed for main agronomic properties: granulometry, organic matter content, cation exchange capacity (CEC), N (%), *C*/*N* ratio, pH, and metal content (Aurea AgroSciences, Ardon, France), and the rest was stored at 4°C for further experiments. Sampled soil consists of a calcareous sandy soil (12.5% CaCO_3_ content and 73.1% sand content, including 65.6% coarse sand) with an organic matter content of 4.7% and a pH_*KCl*_ value of 7.5. All results for the studied parameters are listed in Supplementary Table 3. Metal and metalloid concentrations in the studied soil were monitored in a previous project (AquaNES H2020 project). They were found to be globally low, and these compounds do not represent a major concern on site. CECs are often detected in water samples from the site. A monitoring of the concentrations of several CECs was performed on a 3-year period on water from the site (AquaNES project, H2020). DZ was always found below the detection limit (<20 ng L^–1^). OXA was detected in nearly each point, at every sampling date. OXA concentrations reached 40 ng L^–1^ in a pond upstream of the site filled with water from the golf ponds, and 2,663 ng L^–1^ in the WWTP outlet water.

### Experimental Setup

#### Chemicals and Reagents

Diazepam and OXA standards of high purity grade (>98%) were purchased from Sigma-Aldrich (Steinheim, Germany). Diazepam D5 and oxazepam D5, used as soil extraction standards, were purchased from CIL Cluzeau (Sainte-Foy-la-Grande, France) and Sigma-Aldrich, respectively. Acetonitrile and water with UPLC/MS quality were purchased from Fisher (Illkirch, France). Methanol, phosphate buffer (KH_2_PO_4_), and formic acid were purchased from Sigma-Aldrich (Steinheim, Germany). QuEChERS commercial extraction kits (EN method) were obtained from Agilent Technologies (Santa Clara, PA, United States).

#### Experimental Conditions and Soil Spiking

After sampling, the water contents and water holding capacity (WHC) were determined. Soils were sieved at 5 mm to discard all gravel or plant debris. Then, four sets of soils used for spiking the experiment (dried and crushed, one for each condition) were prepared with a final concentration of 10 mg kg^–1^ of DZ or OXA. These were prepared using stock solutions prepared in HPLC-grade acetone. After adding the DZ or OXA, spiked soils were left for 24 h at room temperature under an extractor hood to allow the acetone to evaporate and were then homogenized by mixing. The concentrations in these spiked soils were checked and were 9.47 mg kg^–1^ DW for OXA abiotic condition, 8.98 mg kg^–1^ DW for OXA biotic condition, 8.42 mg kg^–1^ DW for DZ abiotic condition, and 8.12 mg kg^–1^ DW for DZ biotic condition.

The experiment was then set up by adding the same aliquot of these dry spiked soils to fresh soils by distributing the powder as homogeneously as possible to each of the 82 microcosms (41 DZ and 41 OXA). The initial concentration of both DZ and OXA was quantified in microcosms by using QuEChERS extraction, as described below. These concentrations were 1.96 ± 0.10 mg kg^–1^ DW for the DZ biotic condition, 2.05 ± 0.04 mg kg^–1^ DW for the DZ abiotic condition, 1.71 ± 0.17 mg kg^–1^ DW for the OXA biotic condition, and 1.88 ± 0.06 mg kg^–1^ DW for the OXA abiotic condition. It must be noted that DZ was not detected (<LQ) in the native soil, and OXA was found at a concentration of 25 μg kg^–1^ DW soil. This native concentration was considered as negligible compared to the added OXA. To obtain a water content equal to 70% of the maximum soil WHC, deionized water was added to each microcosm and soils were homogenized again.

The experiment was conducted in triplicate for 97 days. Microcosms were sacrificed at nine sampling dates, after 0, 8, 14, 21, 33, 47, 61, 76, and 97 days of incubation. Sterile controls were performed in duplicate for seven of the sampling dates (controls at 8 and 21 days were not performed) with soils sterilized by gamma ray ionization, in the same conditions as the biotic samples. The experimental setup is presented in Supplementary Figure 1. Measurements of water moisture were also performed during the course of the experiment to ensure its stability at values approximately 70% WHC.

### Chemical Analyses

#### QuEChERS Extraction of Benzodiazepines

Five grams of sieved soil was weighed in a 50-ml polypropylene extraction tube and spiked with 80 μl of a solution of isotopically labeled compounds (internal standards) and then vortexed. The soil was then mixed with 8 ml of phosphate buffer (KH_2_PO_4_) and 10 ml of acetonitrile containing 5% formic acid. The mixture was agitated with an Agitax shaking device (AgytaxLab, Madrid, Spain) for 30 s, after which QuEChERS salt was added. The tube was again agitated for 60 s and finally centrifuged (5 min, 4,000 rpm). The supernatant was entirely collected (about 10 ml per sample) and weighed. For each experimental replicate, three extractions were performed (three samples per microcosm). The three supernatants of these three extractions were combined, yielding a total extraction volume of approximately 30 ml. This was then evaporated down to 12 ml and kept at −20°C prior to analysis by UPLC-MS/MS.

Performance characteristics of the QuEChERS method for the selected pharmaceutical compounds were evaluated on the same soil as the one used in the microcosms. The recovery rate was calculated for each compound by spiking the soil at about 30 μg kg^–1^ of dry soil (in triplicate) and then processing the extraction as described above. Matrix effects were studied by first extracting the sample (in triplicate, and with the procedure described above) and then spiking the total extracted sample at about 30 μg kg^–1^ of dry soil for each compound (i.e., about 120 μg L^–1^ in total supernatant).

#### Quantification of Benzodiazepines

The analytical method was established to quantify a list of 24 pharmaceutical compounds in soil extracts. Chromatographic separation was performed with a Waters Acquity UPLC using a 150 mm × 2.1 mm UPLC-HSST3 C18 1.8 μm column (Waters, Guyancourt, France). Ten microliters of soil extract was injected. The mobile phase was composed of solvent A (0.007% formic acid in water) and solvent B (0.007% formic acid in acetonitrile) at a constant flow of 0.4 ml/min. The initial amount of B was 0% during 0.75 min and linearly increased to 100% in 15 min, followed by a 1-min isocratic mode and returned quickly to the initial conditions. These conditions were maintained for 2.5 min; the total run time was 19 min. Column and autosampler temperatures were maintained, respectively, at 45°C and 10°C. For more details, see Togola et al. (2014).

Mass spectrometry was performed on a triple quadripole (Quattro Premier XE, Waters) in Multiple Reaction Monitoring (MRM) fitted with an electrospray ionisation (ESI) interface and controlled by MassLynx software. Mass spectrometry conditions were previously described (Togola et al., 2014). Briefly, positive and negative polarity modes were used simultaneously during the same analytical run.

The limits of quantification for DZ and OXA were evaluated at 4 μg kg^–1^ of fresh soil. The half-life dissipation time (DT50) for DZ and OXA was calculated with a first-order kinetic model.

### Microbial Community Analyses

#### Extraction and Quantification of Soil DNA

DNA extractions were performed on soils from each condition at all of the sampling times, using the FastPrep DNA Spin Kit for soils (MP Biomedicals) following the manufacturer’s instructions, at a speed of 5 m s^–1^ for 30 s, on 0.7–1 g of humid soil sample and eluted in 100 μl of DES. Total extracted DNA was quantified with a Quantus Fluorometer (Promega) and stored at −20°C prior to further analysis.

#### qPCR of 16S rRNA and 18S rRNA Genes

Quantification of the bacterial 16S rRNA and fungal 18S rRNA gene copies was performed on all DNA samples by qPCR using a CFX Connect^TM^ Real-Time PCR Detection System (Bio-Rad, France). The primers used were 341F (3′-CCTACGGGAGGCAGCAG-5′) and 515R (3′-ATTACCGCGGCTGCTGGCA-5′) (Lopez-Gutierrez et al., 2004) for 16S rRNA genes, and primers FF390 (5′-CGATAACGAACGAGACCT-3′) and FR1 (5′-A5CCAT TCAATCGGTA5T-3′) for 18S rRNA genes (Vainio and Hantula, 2000). qPCR reactions were performed in a total volume of 20 μl, with a mix containing 7.6 μl of sterile, nuclease- and nucleic acid-free water (MP Biomedicals, CA, United States), 10 μl of SSO Advanced Universal SYBR Green Supermix (Bio-Rad), 400 nM (16S rRNA genes) or 500 nM (18S rRNA genes) of each primer, 100 ng of T4 GP32 (16S rRNA genes), and 2 μl of template DNA (0.1–5 ng⋅μl^–1^). Sterile, nuclease- and nucleic acid-free water was used instead of DNA in negative controls. For 16S rRNA gene, PCR reactions were performed as follows: 3 min at 95°C, followed by 35 cycles: 30 s at 95°C/30 s at 60°C/30 s at 72°C/30 s at 80°C. For 18S rRNA gene, PCR reactions were performed as follows: 30 s at 95°C/30 s, 40 cycles: 15 s at 95°C/30 s at 50°C/30 s at 72°C/30 s at 80°C. At the end of the qPCR, an additional step rising from 60 to 95°C at 0.5°C/s was applied for melt curves generation.

A calibration curve was obtained from serial dilutions of a known quantity of linearized plasmids containing known copy numbers of 16S rRNA or of 18S rRNA gene. All samples, controls, and standards were analyzed in duplicate. Results were reported as number of gene copies per gram of dry soil. Generation of a specific PCR product was confirmed by melting curve analyses and agarose gel electrophoresis.

#### Next-Generation Sequencing (16S rRNA Gene and ITS2)

Microbial diversity was evaluated by Illumina MiSeq sequencing of the 16S rRNA gene for bacteria and archaea and ITS2 region for fungi. Sequencing was performed on 19 samples: native soil (FR4N), and three sampling dates, in triplicates, for DZ and OXA microcosms. Chosen dates were T0, T14, and T97 for microcosms corresponding at 0, 14, and 97 days after benzodiazepines spiking, respectively. The native soil corresponded to the fresh soil that was used for microcosms, before spiking.

Amplicon libraries were constructed by INRAE Transfert (Toulouse, France). Briefly, a portion of the 16S rRNA gene (V4–V5 region) was amplified using the barcoded, universal primer set 515WF—GTGYCAGCMGCCGCGGTA/928WR—CCCCGYCAATTCMTTTRAGT (Wang and Qian, 2009). Concerning ITS2 gene for fungi, a portion of the ITS2 gene was amplified using the specific primer set w404 CTTTCCCTACACGACGCTCTTCCGATCTTGTGARTCATCG AATCTTTG/w40 GGAGTTCAGACGTGTGCTCTTCCGATCT TCCTCCGCTTATTGATATGC (Hadziavdic et al., 2014). PCR reactions were performed using the AccuStart II PCR ToughMix kit, followed by cleaning (HighPrep PCR beads, Mokascience), and pooled triplicates were submitted for sequencing on Illumina MiSeq instrument at GeT-PlaGe (Auzeville, France). Fastq files were uploaded to the Galaxy web platform (Sygenae), and the public server at usegalaxy.org was used to analyze the data, with the FROGS analysis platform (Escudié et al., 2017). For 16S rRNA gene sequences, affiliations were obtained using the Silva 138_16S database. For ITS2, Unite_Fungi_8.0_18112018 reference database was used for OTUs affiliation. The affiliation tables and sequence counts can be found in Supplementary Tables 1, 2. The sizes of reads for R1 and R2 (forward and reverse) were 250 bp. For trimming, the FROGS Pre-process merging, denoising, and dereplication tool was used (Galaxy Version r3.0-3.0). Mismatch rate was set to 0.1 (maximum rate of mismatch in the overlap region of R1 and R2 in consensus sequences), and merge calculations were performed using Flash software for an expected amplicon size of 413 bp (min 340 bp and max 450 bp) for 16S rRNA genes, and 389 bp (min 267 bp and max 511 bp) for highly variable ITS2 region extracted using the FROGS ITSx tool (Galaxy Version r3.0-1.0). Then, clustering was performed using FROGS Clustering swarm amplicon sequence clustering tool (Galaxy Version r3.0-1.4) with denoising. The aggregation distance clustering was set to a maximum of 3 bp for inclusion into clusters. To remove the PCR chimera in each sample, the FROGS Remove chimera tool was used (Galaxy Version r3.0-7.0). This tool is based on the VSEARCH tool for chimera detection and removal (Rognes et al., 2016). Alpha diversity indices were calculated from the NGS data. The first index corresponds to the observed number of OTUs in data from 16S rRNA gene and ITS2 region sequencing. The Chao1 index estimates the unobserved OTUs by correcting values with OTUs that are observed only one or two times in data (correction for the rarest OTUs). The Shannon and Inverse Simpson indices give an indication of the specific richness in the samples, by linking the number of species with the specific abundance of these species in the different samples. Tax4Fun data were also generated from FROGS platform, and potential metabolic pathways were recovered by comparison of the 16S rRNA gene sequences with the KEGG database (Aßhauer et al., 2015). For the 19 samples, only 1.79–2.91% of OTUs could be mapped to KEGG organisms. All 16S rRNA gene sequences and ITS2 sequences were submitted to European Nucleotide Archive (ENA) and are available under accession numbers ERS7290419 (SAMEA9567684) to ERS7290437 (SAMEA9567702). The whole project is referenced under accession number PRJEB47338.

### Statistical Analyses

Statistical analyses were performed on XLStat software v2013.3.2 (Addinsoft, 2020). Alpha and beta diversity calculations were performed using FROGS platform. Co-occurrence networks were built using Cytoscape software v3.8.0 with CoNet add-on.

## Results

### Evolution of Benzodiazepine Concentrations in Microcosms

**FIGURE 2:**
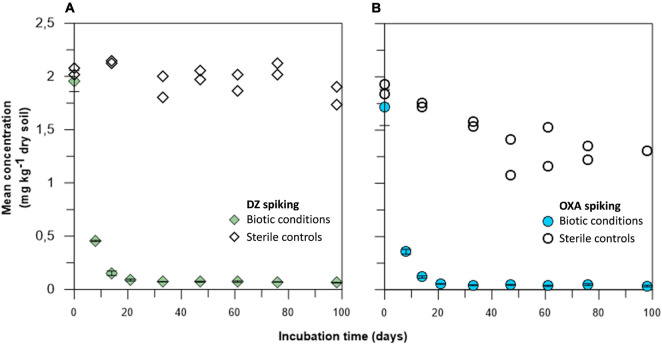
Diazepam (DZ) **(A)** and oxazepam (OXA) **(B)** biodegradation kinetics over a 97-day period. Values for biotic conditions are presented as mean values of triplicates with standard deviations, and points for sterile controls are presented as sole values for both duplicates (see Figure 3 for details).

Concentrations of DZ and OXA over time, in the different microcosms and their sterile controls, were monitored on nine sampling dates (Figure 2). It firstly appeared that concentrations in the sterile controls treated by gamma-ray remained high over time, especially for DZ, indicating that sterilization was effective. Consequently, the observed results in microcosms were linked to biodegradation of DZ and OXA. In the biotic soil microcosms, biodegradation or biotransformation occurred both for DZ and for OXA very quickly: DT50 for DZ, based on a first order degradation model, was 3.9 and 3.6 days for OXA. After 20 days, barely all the spiked benzodiazepines were dissipated [95.5% ± 0.5% (*n* = 3) and 96.9% ± 0.2% (*n* = 3) for DZ and OXA, respectively] (Figure 2), and only trace concentrations remained, whereas concentrations remained stable in sterile controls at approximately 1.5–2 mg kg^–1^ DW soil. OXA concentrations tended to decrease slightly over time in sterile controls. It is not impossible that gamma-ray treatment did not destroy all microorganisms, especially sporulating microorganisms. Microorganisms in sporulated form during the sterilization could have grown later, explaining a slight biodegradation in controls especially for OXA conditions. It is also possible that abiotic degradation of OXA occurred during the experiment. Samples were stored in the dark, and benzodiazepines are resistant to photodegradation, but other abiotic reactions could have led to the observed OXA concentrations decrease in sterile controls. Results are, however, globally satisfying in sterile controls, and significantly different compared to biotic conditions. These results highlight a good removal rate of the two studied benzodiazepines in the studied soil, rather by biodegradation or biotransformation of the mother molecules.

### Bacteria and Archaea Dynamics Based on 16S rRNA Gene Abundance and Diversity

#### Initial Bacteria and Archaea Soil Diversity

**FIGURE 3:**
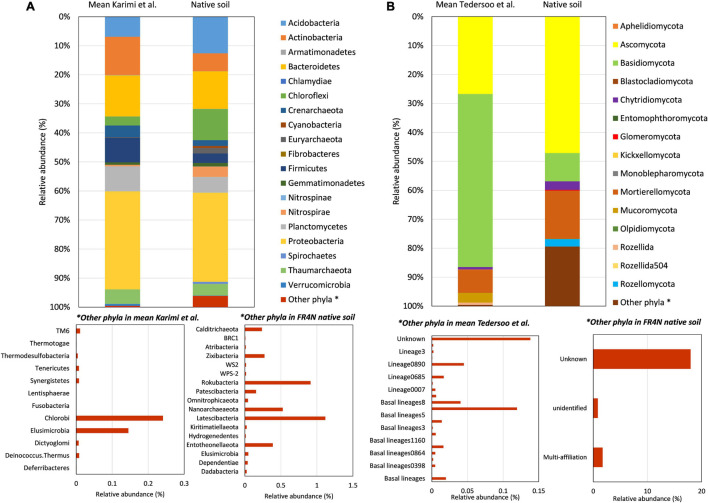
Comparison of microbial diversity at the phylum level for **(A)** bacteria and archaea between FR4N soil and data from Karimi et al. (2018), and **(B)** fungal diversity between FR4N soil and data from Tedersoo et al. (2014). Data from Karimi et al. (2018) correspond to mean diversity in 2,173 soil samples from France and data from Tedersoo et al. (2014) correspond to mean values in a selection of 127 samples corresponding to representative couples of soil/climate that can be found in France (i.e., temperate coniferous forests, temperate deciduous forests, grassland, and shrubland and Mediterranean soils).

Prokaryote community diversity was compared to the average diversity observed among 2,173 studied soils in France (Karimi et al., 2018). At the phylum level, differences were observed between the native FR4N soil and the mean diversity described in Karimi et al. (2018) for soils in France (Figure 3A). Among dominant phyla, Acidobacteria and Chloroflexi were approximately two and three times more represented, respectively, in FR4N soil compared to mean values in French soils. Although they are not highly represented, Chlamydiae, Cyanobacteria, Euryarchaeota, Spirochaetes, Nitrospinae, and Nitrospirae are, respectively, 10, 16, 422, 229, 6, and 7 times more represented in the FR4N soil compared to mean values in French soils. On the contrary, among dominant phyla, Actinobacteria, Crenarchaeota, and Firmicutes were approximately two to three times less represented in the FR4N soil compared to the mean values in French soils. Verrucomicrobia were approximately six times less represented. Among the rarest phyla, Chlorobi (0.25% in the soils of France) are absent from the studied soil. On the contrary, Calditrichaeota, Zixibacteria, Rokubacteria, Nanoarchaeaeota, Latescibacteria, and Entotheonellaeota represented 0.25–1% of the global studied soil diversity, whereas they are not described in the main phyla found in French soils (Karimi et al., 2018).

#### Evolution of Bacterial and Archaeal Abundance

Bacterial and archaeal abundances were evaluated by quantification of the number of 16S rRNA gene copies per gram of DW soil (Supplementary Figure 2A for DZ conditions and Supplementary Figure 2B for OXA conditions). In both DZ and OXA conditions, the abundance of 16S rRNA gene was significantly lower at T0, maybe due to the toxicity of added contaminants. At T8, values are intermediate, and a plateau is reached after 14 days. It appears that the period between T0 and T14 corresponds to a growth phase and that the bacterial and archaeal communities remain stable after 14 days up to the end of the monitoring at 97 days. However, they remained slightly less abundant compared to the native soil.

#### Evolution of Bacterial and Archaeal Diversity

As shown by the total number of OTUs and Chao1 index, biodiversity increased after benzodiazepine addition, compared to the native soil, and did not significantly change after (Supplementary Figures 3A,B; Kruskal–Wallis test with Conover–Iman comparison, *p* < 0.05, Supplementary Figure 3). Results are comparable between DZ and OXA. This increase is overall low (approximately 50–100 OTUs compared to the native soil), but significant, especially looking at the “corrected” Chao1 index (Supplementary Figure 3B). The total number of OTUs is maximum at T14 (Supplementary Figure 3A), when the majority of DZ and OXA were biodegraded, and it tends to decrease at T97.

**FIGURE 4:**
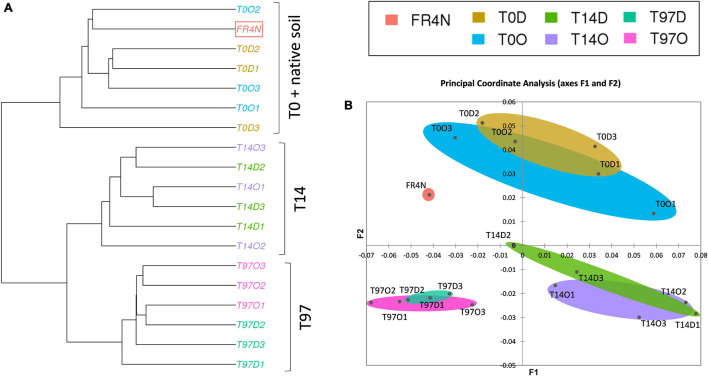
**(A)** Ward D2 linkage clustering tree of the different samples based on the 16S OTUs repartition and beta diversity values (Bray–Curtis dissimilarities) and **(B)** principal coordinate analysis (PCoA) based on Bray–Curtis dissimilarity distances between the different samples.

Shannon and Inverse Simpson indices (Supplementary Figures 3C,D) were lowest at T0 and highest (and significantly different, Kruskal–Wallis test with Conover–Iman comparison, *p* < 0.05) at T14, for both DZ and OXA. They then tended to decrease to recover values comparable to T0 and the native soil at T97. A ward linkage-clustering tree (beta diversity; Figure 4A) highlighted three distinct groups: (i) T0 and native soil, (ii) T14, and (iii) T97. Bacterial and archaeal communities were consequently similar in native soil and at T0 (abundance was lower in T0 samples but the bacterial and archaeal diversity were similar), and they changed at T14 and T97. Biodiversity was more similar at T14 and T97 compared to T0, highlighting a change due to the addition of benzodiazepines. Small differences were observed between DZ and OXA conditions, independently from the sampling date. The PCoA analysis (Figure 4B) also revealed the three-group repartition of OTUs, for T0, T14, and T97 samples, respectively.

**FIGURE 5:**
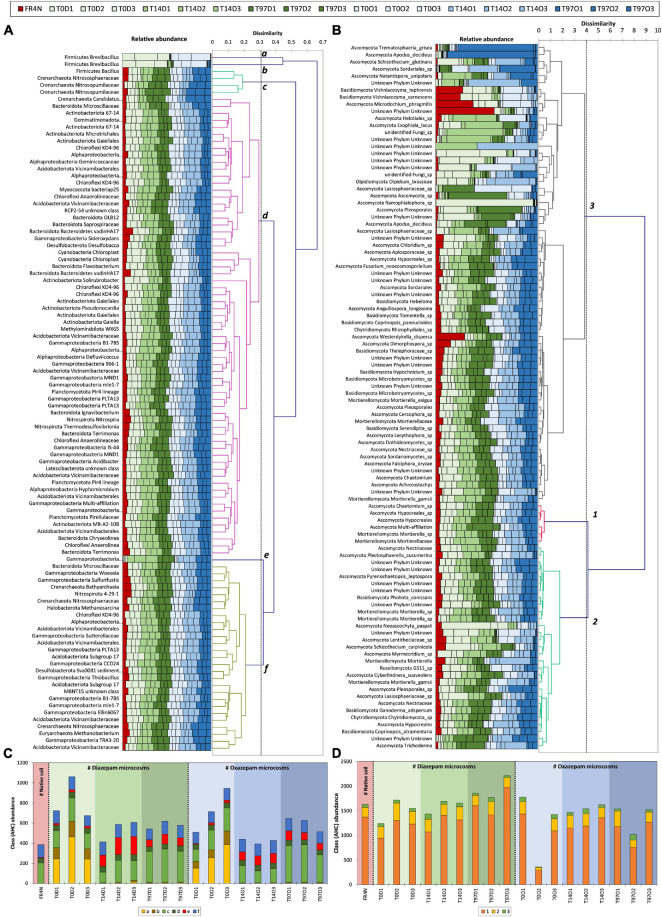
Agglomerative Hierarchical Clustering evaluated from Bray–Curtis distances with proportional link as aggregation method for the 100 most abundant OTUs (sum on the 19 samples), for **(A)** bacterial and archaeal OTUs and **(B)** fungal OTUs. Panel **(C)** corresponds to the histogram presenting the repartition of the six different classes defined by AHC for bacterial and archaeal OTUs and **(D)** the three classed defined by AHC for fungal OTUs.

Evolution of the relative abundance of the 100 most abundant OTUs (sum for the 19 samples) is shown and agglomerative hierarchical clustering (AHC) of these OTUs was performed and is presented in Figure 5A. The six classes defined by the AHC are also presented in Figure 5C. Six evolution patterns could be highlighted, corresponding to the six classes defined for bacterial and archaeal OTUs. Classes a and b (yellow and brown on Figure 5C) correspond to OTUs that are much more abundant at T0 compared to the other sampling dates, in DZ and OXA conditions. They are also more abundant at T14 and T97 compared to the native soil, but to a lower extent. These classes correspond to Firmicutes, and more precisely to two different OTUs of the *Brevibacillus* genus. Class c corresponds to OTUs that are relatively more abundant at T97. This class corresponds to three archaeal OTU from the Crenarchaeota phylum and one bacterial OTU from the Firmicutes phylum (*Bacillus* genus); two of the archaeal OTUs belong to the Nitrosopumilaceae order, and were unknown or multi-affiliated at the genus taxonomic level, and the other one belongs to the Nitrososphaeraceae order. Classes d and f correspond to the high majority of OTUs (93 OTUs over the 100 most abundant). The relative abundance of these 93 OTUs remained stable during the whole experiment. Lastly, class e corresponds to an OTU that was significantly more abundant at T14 compared to the native soil and T0, and more abundant at T97 but in a lower extent. This OTU belongs to Gammaproteobacteria, *Hydrogenophaga* genus. It appears that differences concern a low proportion of the detected OTUs and are limited to OTUs belonging to the Crenarchaeota, Firmicutes, and Gammaproteobacteria phyla.

Overall, few differences were found between DZ and OXA conditions, and the structure of the bacterial and archaeal communities was not deeply modified, indicating little effect with the addition of these contaminants.

### Fungal Biodiversity Dynamics Based on 18S rRNA Gene Abundance and ITS2 Region Sequencing

#### Initial Fungal Diversity

Contrary to prokaryotes, data for fungal diversity in French soils are not available. However, Tedersoo et al. (2014) published a worldwide repartition of soil fungal diversity. Samples corresponding to the climates/environments that can be found in France (temperate coniferous forests, temperate deciduous forests, grassland, scrubland, and Mediterranean soils, for 127 samples) were selected and compared to the fungal diversity in the FR4N soil (Figure 3B). In comparison with bacterial and archaeal diversity, larger differences were observed. Indeed, if classically Basidiomycetes are dominant in such soils, they were about six times less represented in the FR4N soil. On the contrary, Ascomycota and Mortierellomycota were two to three times more represented in the FR4N soil. Among phyla that are not highly represented, Chytridiomycota and Glomeromycota were 3.5 and 5.3 times more represented, and the Rozellomycota phylum was about 5,000 times more represented in the FR4N soil. Monoblepharomycota, Olpidiomycota, and Aphelidiomycota phyla were detected in the FR4N soil, even if they are not reported in Tedersoo et al. (2014). On the contrary, Rozellida phylum was not detected in the FR4N soil.

#### Evolution of Fungal Abundance

Fungal abundance in soils was monitored by quantifying the 18S rRNA gene. The same trend as for bacterial abundance was highlighted. Indeed, a lower fungal abundance was observed at the beginning of the experiment, and this abundance then increased up to a plateau reached after 14 days of incubation (Supplementary Figure 4). The fungal abundance remains, however, lower compared to the native soil, with approximately one log abundance difference. Differences between DZ and OXA conditions are low.

#### Evolution of Fungal Diversity

**FIGURE 6:**
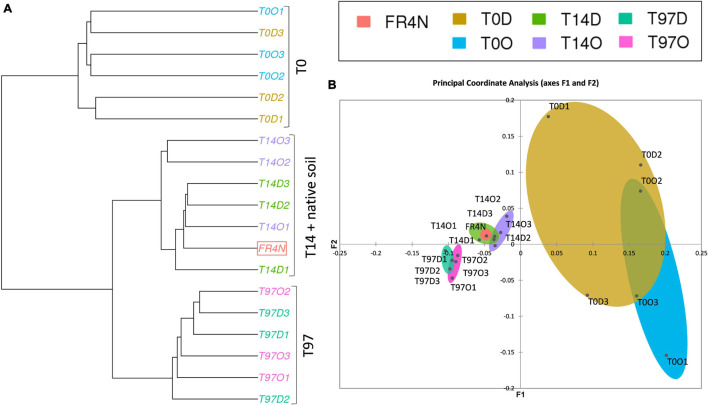
**(A)** Ward D2 linkage clustering tree of the different samples based on the fungal OTU repartition and beta diversity values (Bray–Curtis dissimilarities) and **(B)** principal coordinate analysis (PCoA) based on Bray–Curtis dissimilarity distances between the different samples.

Results for fungi were different compared to bacteria and archaea ones. Indeed, for alpha diversity indices, compared to the native soil, the number of observed fungal OTUs was significantly lower at T0 (Supplementary Figures 5A,B). The difference was less marked regarding specific richness of OTUs (Supplementary Figures 5C,D). Concerning the number of observed OTUs and Chao1, values were again comparable to the native soil at T14, as they were significantly higher compared to T0 (Kruskal–Wallis test with Conover–Iman comparison, *p* < 0.05). The Shannon and InvSimpson indices tended to decrease during the experiment up to T97 but no significant difference could be highlighted here. The ward linkage clustering tree of fungal OTUs (beta diversity; Figure 6A) highlighted three distinct groups: (i) T0, (ii) T14 and native soil, and (iii) T97. Results thus differed from those for bacteria and archaea. Indeed, whereas T0 was similar with the native soil for bacterial and archaeal OTUs, here the native soil seems to be closer to the T14 samples. This is coherent with results from alpha diversity indices. Indeed, the impact on fungi seems to be higher right after benzodiazepines spiking. Consequently, samples from T0 are more different compared to native soil, T14 and T97. The PCoA analysis (Figure 6B) also revealed this “three-group” repartition. Samples from T14 and T97 were closer, and T14 was especially close to the native soil. T0 samples were the more different compared to the others, highlighting a possible higher impact of the addition of benzodiazepines for fungi.

#### Identification of Fungi Taxa

Even if biodiversity indices did not show a clear response of the global fungal community to DZ and OXA spiking, some OTUs showed a specific evolution as shown by the AHC on the 100 most abundant fungal OTUs (Figures 5B,D). Trends are overall more difficult to observe compared to bacteria and archaea, as in some cases, there is a high variability between triplicates. Three classes could, however, be highlighted regarding their evolution during DZ and OXA biodegradation (Figure 5D). Classes 2 and 3 corresponded to the majority of OTUs, 29 and 65 over the 100 most abundant, respectively. These OTUs did not present a high evolution during the degradation kinetics, and values remained overall stable and comparable to the native soil. Class 1 corresponds to six OTUs, for which the observed evolution during biodegradation was different. These six OTUs belonged to two phyla, Ascomycota (four OTUs) and Mortierellomycota (two OTUs). Among Ascomycota, one OTU had multi-affiliation at a higher taxonomic level, two OTUs belonged to the Hypocreales order (unidentified at a higher taxonomic rank), and one was affiliated to the *Chaetomium* genus. Among Mortierellomycota, one OTU belonged to the *Mortierella* genus, and the other was affiliated to the Mortierellaceae family. Both of these six OTUs presented a slightly higher abundance during the biodegradation kinetics experiments and tended to be more present at T97 compared to the native soil (Figure 5D).

### Co-occurrence Between Most Abundant Bacterial, Archaeal, and Fungal Operating Taxonomic Units

**FIGURE 7:**
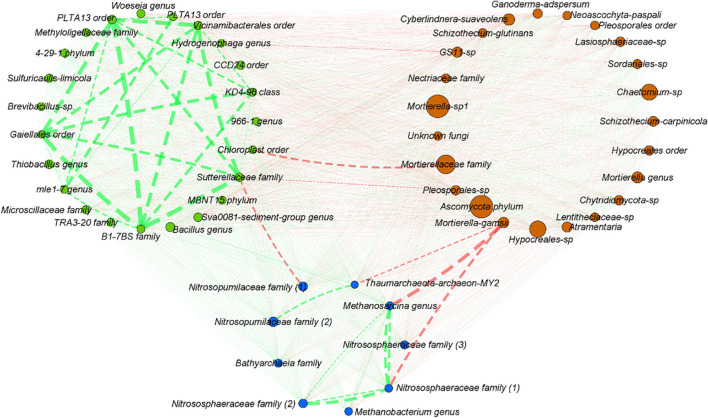
Co-occurrence network between the 23 most abundant bacterial OTUs, the 9 most abundant archaeal OTUs, and the 23 most abundant fungal OTUs. Network was built using Pearson, Spearman, and Bray–Curtis analyses, both for significance thresholds <0.05. Co-occurrences (co-presence marked in green, mutual exclusion marked in red) are highlighted if significant for at least two of the three previously cited methods. The line width is proportional to the weight of the correlation, ranged from –0.828 to –0.710 for mutual exclusion (red) and from 0.881 to 0.981 for co-presence (green). The number of OTUs represented was chosen arbitrarily after many assays to make the network more readable as possible.

To explore potential interactions between bacteria, archaea, and fungi, a co-occurrence network was built with the 23 most abundant bacterial OTUs and fungal OTUs and 9 most abundant OTUs for archaea (Figure 7). No significant mutual exclusion could be highlighted between the selected bacterial OTUs. Overall, seven significant mutual exclusions were highlighted, one between bacteria and archaea, three between bacteria and fungi, and three between archaea and fungi. Mutual exclusions were observed (i) between the Sutterellaceae and Nitrosopumilaceae families and (ii) an OTU affiliated to the Pleosporale order; (iii) between the Chloroplast order and Mortierellaceae family; (iv) between *Mortierella* genus (close from *Mortierella gamsii*) and Thaumarchaeota archaeon MY2; (v) between *Methanosarcina* genus and (vi) one OTU from the Nitrososphaeraceae family; and (vii) between the *Hydrogenophaga* genus and an OTU from the GS11 fungal order (Figure 7). Co-occurrences were more abundant compared to mutual exclusions, but within kingdoms only. Indeed, no significant co-occurrences were highlighted between taxa of bacteria/archaea/fungi. High co-occurrence of the 23 most abundant bacterial OTUs was observed, not for every OTU but for the majority of them. For archaea, co-occurrence was especially observed between Nitrososphaeraceae family and *Methanosarcina* genus, and between one OTU from the Nitrosopumilaceae family and another one close to Thaumarchaeota archaeon MY2. Concerning fungi, no co-occurrence between the 23 selected OTUs could be highlighted.

### Exploration of the Evolution of Metabolic Pathways Using Tax4Fun

Only approximately 2% of bacterial and archaeal OTUs could be mapped to KEGG pathways; fungi were not considered in this approach. Nevertheless, a total of 3,554 metabolic pathways were detected in the data from the OTUs affiliation. These were compared between samples from microcosms during benzodiazepine biodegradation and the native soils. Among the 3,554 pathways, some were enhanced compared to the native soils (the majority), and some declined. Indeed, among the 3,554 characterized pathways, between 44.9 and 49.4% were enhanced by at least 10% compared to the native soil, 9.7–21.1% by 50%, 3.3–8.6% by 100%, 0.1–1.4% by 500%, and 0.06% to 0.6% by 1,000% (20 pathways). The latter were five transferases, five Oxidoreductases, seven hydrolases, one lyase, and three proteins linked to ADP/ATP metabolism (Supplementary Table 1). Most of them were linked to redox reactions and to ADP–ATP cycles, highlighting an increase in microbial global activity. A few more functions were highlighted in DZ conditions compared to OXA, but overall results are close between the two molecules, suggesting that the processes involved in their biodegradation may be similar.

## Discussion

The objectives of this study were to assess the capacity of soil microbial communities (bacteria, archaea, and fungi) from a study site periodically flooded by water from a WWTP, to resist and potentially degrade benzodiazepine molecules, especially DZ and OXA when introduced into the soil. The study was based on a soil sampled in a soil aquifer treatment (SAT) context, where water previously treated in a WWTP are injected in the SAT area. The aim was to focus on two recalcitrant CECs, DZ, and OXA, and simulate an input in the studied soil to study their behavior, including microbial biodegradation.

### Soil Biodiversity in a Soil Aquifer Treatment Context at Agon-Coutainville

It was previously shown that biodiversity in river could be affected by WWTP effluents (Drury et al., 2013; Blunt et al., 2018). Our study highlights that it may also be the case for soils in a SAT context. At the prokaryotic phylum level, several differences were found between the study soil and the mean diversity of French soils as described by Karimi et al. (2018) (Figure 3A). Among dominant phyla, Acidobacteria and Chloroflexi were approximately two and three times more represented, respectively, in our soil. Acidobacteria and Chloroflexi phyla contain diverse metabolic profiles, including strong links with carbon cycle. Acidobacteria are very commonly found in different environments including soils (de Chaves et al., 2019). Genomic and physiological characteristics of Acidobacteria were recently reviewed (Kielak et al., 2016). They concluded that many gaps still exist to fully understand the functional role of Acidobacteria in the soil C degradation process. However, it was also shown that both microbial C degradation processes and acidobacterial communities can be affected by soil management (de Chaves et al., 2019), suggesting a potential impact of soil use and characteristics on the abundance of Acidobacteria. The phylum Chloroflexi is composed of diverse groups of organisms that include anoxygenic photoautotrophs, aerobic chemoheterotrophs, thermophilic organisms, and anaerobic organisms that obtain energy through reductive dehalogenation of organic chlorinated compounds (Gupta, 2013). Other phyla such as Chlamydiae, Cyanobacteria, Euryarchaeota, Spirochaetes, Nitrospinae, and Nitrospirae were found to be overrepresented compared to mean values in French soils. Actinobacteria, Crenarchaeota, and Firmicutes were approximately two to three times less represented, and Verrucomicrobia were approximately six times less represented. The identity of the rarest phyla (such as Calditrichaeota, Rokubacteria, Nanoarchaeota, Latescibacteria, Chlorobi, and Elusimicrobia) was also found to be different. The repeated inputs of organic substances from WWTP in the studied soil could explain their higher abundance compared to “classical” abundances in soil. Differences could also come from the microbial inputs from the WWTP biological treatment. Indeed, Drury et al. (2013) showed that the input of WWTP effluent modified the nature of bacterial communities in water and sediments (Drury et al., 2013), especially due to the input of contaminants from WWTP.

Concerning fungi in the studied soil (Figure 3B), Basidiomycetes were found to be about six times less represented, whereas Ascomycota and Mortierellomycota were two to three times more represented. Basidiomycetes are generally described as the principal fungal phylum in soils in terms of abundance (Tedersoo et al., 2014). In our case, Ascomycota were the dominant phyla. Fungi are heterotrophic organisms; they need organic carbon from external sources to live. With soil pH and plant cover (Wubet et al., 2012), total organic carbon (TOC) content is generally described as the main parameter controlling the abundance and diversity of soil fungal communities (Tedersoo et al., 2014; Liu et al., 2015, 2018a,b). The specificity of the studied soil, with high organic matter, and contaminants, contains all the ingredients for fungal development. Liu et al. (2015) showed that the relative abundance of Basidiomycota was negatively correlated (*r* = −0.600) to the soil carbon content in a selection of 25 soil samples (Liu et al., 2015). For Ascomycota, a high carbon content was more positively correlated to Ascomycota abundance. Our soil contains 4.7% of organic matter, corresponding to 47 g kg^–1^ dw soil, which is a quite high OM content. This is possibly the reason why Basidiomycota were found to be less abundant, and Ascomycota more abundant compared to classical values. Moreover, it was shown that Ascomycota were the main representatives of the fungal community in a soil contaminated by PAHs and trace metals (Bourceret et al., 2016). The input of (organic) contaminants from WWTP effluents could then also explain the dominance of Ascomycota. Among phyla that are not highly represented classically, significant differences could also be highlighted. Rozellomycota were about 5,000 times more represented in our soil compared to mean values from Tedersoo et al. (2014). Rozellomycota constitutes the basal lineage of the kingdom of fungi. They predominately feed by osmotrophic nutrition (Tedersoo et al., 2018), meaning that they feed on dissolved substances. The high inputs of dissolved substance from WWTP could explain why Rozellomycota are 5,000 times more represented compared to classical soils. They could also take part in the degradation of organic micropollutants. Presently, little is known about Rozellomycota, but our results suggest that they may be used as a bioindicator to characterize an environment impacted by WWTP effluents or sewage sludges.

### Biodegradation/Biotransformation of Diazepam and Oxazepam

In our study, sorbed DZ and OXA were extracted during the QueChers procedure; biodissipation as well as extraction procedure could be attested thanks to comparison with sterile gamma-ray ionized controls, where concentrations remained stable during the experiment. It is thus possible to affirm that a biological process occurred, explaining the observed dissipation of DZ and OXA in the microcosms. Our dissipation kinetics results are highly more rapid compared to most of the results previously described. Indeed, in the studied soil, DT50 was 3.9 and 3.6 days for DZ and OXA, respectively, and after 97 days (and even almost after 20 days), 95.51% ± 0.49% and 96.87% ± 0.22% of the spiked DZ and OXA, respectively, were dissipated due to biological activity of fungi, bacteria, and/or archaea.

The biodegradability of DZ and OXA is often described as very limited in the environment as well as in lab-scale biological experiments (Ternes et al., 2004; Redshaw et al., 2008; Suarez et al., 2010; Klaminder et al., 2015; Greskowiak et al., 2017; Min et al., 2018). They were also shown to be resistant to biological treatments in WWTPs (Falås et al., 2016), explaining their inputs in the studied soil. Indeed, most of the data on the efficiency of WWTP treatments concerns DZ and shows that (i) less than 10% is removed during conventional biological treatment, (ii) the efficiency seems to be higher during anaerobic treatment (10–50%), and (iii) the removal rate is mainly related to sorption rather than degradation (Ghattas et al., 2017). Similarly, OXA is described as persistent in both aerobic and anaerobic treatments (Patterson et al., 2010; Kosjek et al., 2012). Some degradation pathways were, however, highlighted, such as demethylation (Helbling et al., 2010), but an efficient biodegradation in the environment has not yet been reported. Different values available in the literature for DZ and OXA biodegradation are listed in Table 1. Some researchers found that DZ was not directly degradable by bacteria, especially when other carbon sources more easily accessible are available, which is the case in soils (Tappin et al., 2014). A mixed microbial culture from activated sludge treatment has also been shown to demethylate DZ to form 7-chloro-5-phenyl-3H-1,4-benzodiazepin-2-one (Helbling et al., 2010). The same culture also formed a mono-hydroxylated DZ derivative, but the exact position of the hydroxyl group was not further specified. Redshaw et al. (2008) investigated the susceptibility to microbial degradation of a selection of CECs, including DZ and OXA, in short-term (60 days) bacterial liquid cultures derived from sewage sludge amended soil. They highlighted that DZ was not degraded or transformed after 60 days, but up to 40% OXA was removed in liquid cultures (Table 1). Kosjek et al. (2012) compared the reports of PBT profiler^2^ (21.10.2011) with the results of their WWTP pilot, consisting of a succession of 4-L units in open circuit under oxic and then anoxic conditions. According to pbtprofiler.net, DZ and OXA are not persistent in water (DT50 < 60 days). These molecules are moderately persistent in soils (DT50 = 60–180 days) and very persistent in sediments (DT50 > 180 days). Based on their experience in anoxic bioreactors or oxic–anoxic cascades, the removal of DZ (100 μg L^–1^ continuous feed) was less than 32%. Similarly, the OXA abatement in oxic conditions was less than 24%. On the other hand, removal in anoxic conditions reached 67% after 1 year of feeding the bioreactor, and the authors suggest an effect of biomass adaptation over time to be confirmed by biomass analyses (quantity and diversity). In our soil, a continuous exposure to CECs, including benzodiazepines, by periodic flooding by WWTP water, could have led to a strong adaptation of the soil microbial community. Löffler et al. (2005) showed that DZ was highly persistent with rapid and extensive sorption on sediments, stable in soils, groundwater, and during wastewater treatment. The same study showed that OXA is moderately persistent in the environment with a DT50 and a lower sorption rate on sediments than DZ. In addition, Löffler et al. (2005) did not observe any degradation of DZ to OXA in sediments. Carballa et al. (2006) showed a removal of 38% ± 21% to 60% ± 18% of DZ (depending on temperature) in anaerobic bioreactors simulating a WWTP treatment. An adaptation of the bacterial community in sludge was highlighted, and better yields were obtained with water thermal pretreatment. Suarez et al. (2010) investigated the degradation of CECs in lab-scale experiments using activated sludge reactors under aerobic and anoxic conditions (Suarez et al., 2010). They highlighted that DZ and trimethoprim were highly resistant to biological transformation, and that the degradation rate for studied CECs was highly dependent on the specific microbial species.

**TABLE 1:** Generally described DZ and OXA biodegradation in environmental samples, cultures from environmental sample, or experimental biodegradation studies.

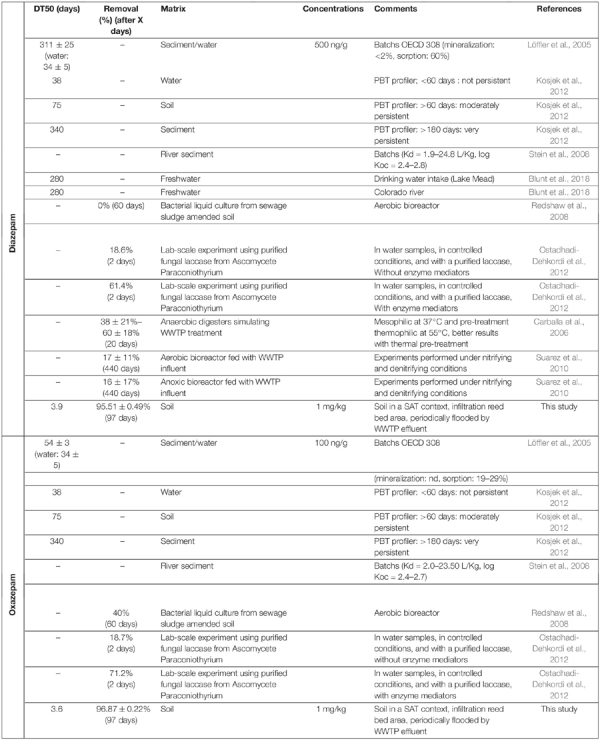

Other studies showed that fungi, especially *via* laccases enzymes, are able to degrade and or transform benzodiazepines. Ostadhadi-Dehkordi et al. (2012) showed a DZ and OXA removal after 48 h of 18.6 and 18.7%, respectively, in lab-scale experiment using purified fungal laccase from Ascomycete *Paraconiothyrium*. They showed that adding enzyme mediators increased the yields up to 61.4 and 71.2% for DZ and OXA, respectively. However, this study was performed in water samples, in controlled conditions, and with a purified laccase (Ostadhadi-Dehkordi et al., 2012). Ambrus et al. (1975) showed that the fungus *Aspergillus niger* J/8 N was able to demethylate DZ to form 7-chloro-5-phenyl-3H-1,4-benzodiazepin-2-one. This study is the first to describe the ability of fungi to biotransform DZ. Rodarte-Morales et al. (2011) studied the potential ability of three white rot fungi strains to degrade pharmaceutical and personal care products (PPCPs) including DZ: an anamorph species of *Bjerkandera* sp. R1, *Bjerkandera adusta*, and *Phanerochaete chrysosporium*. They showed that most of the studied PPCPs were completely degraded except fluoxetine and DZ. DZ recoveries were between 23 and 57% at the end of the experiment, where, for example, carbamazepine was completely degraded.

Overall, DZ and OXA seem to be highly recalcitrant to microbial degradation, but, even if research in this way is still needed, some bacteria or fungi, *via* several degradation pathways, are able to biotransform and/or biodissipate and/or completely biodegrade these compounds. The monitoring of the biodiversity evolution during the biodegradation can then help to find candidates able to perform DZ and OXA biodegradation.

### Bacterial and Archaeal Candidates for Biodegradation of Diazepam and Oxazepam

**FIGURE 8:**
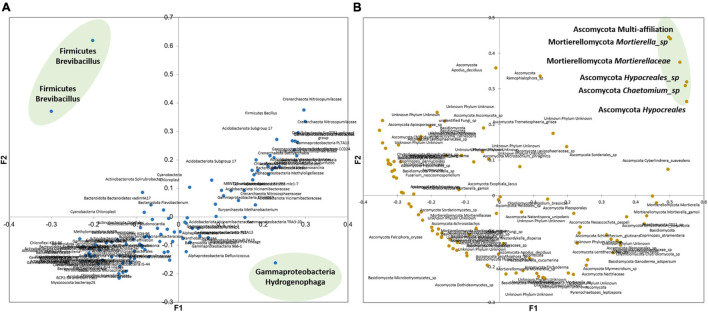
Principal coordinate analysis built from Bray–Curtis dissimilarity matrix of the 100 most abundant (A) bacterial and archaeal OTUs and (B) fungal OTUs. The OTUs of interest are in bold.

As shown in Figure 8A, the main bacteria showing a specific behavior during DZ and OXA biodegradation are two OTUs from the *Brevibacillus* genus and one from the *Hydrogenophaga* genus. *Brevibacillus* has been proposed as a biological tool by Panda et al. (2014) especially for biotechnological applications due, for example, to their ability to biodegrade low-density polyethylene, to act as a candidate bio-control agent, and as a tool for structural and functional biology (Panda et al., 2014). *Brevibacillus parabrevis* has been shown to degrade low-density polyethylene (LDPE), a recalcitrant thermoplastic made from petroleum, by forming a biofilm on LDPE surface, or with granular LDPE as sole carbon source and without nitrogen (Pramila et al., 2012). Also, *Brevibacillus brevis* has been described as able to biodegrade triphenyltin (TPT), a highly toxic endocrine disruptor used as ingredient for antifouling products and fungicides (Ye et al., 2013). Yang and Lee (2007) highlighted the ability of *Brevibacillus* sp. strain P-6 to degrade phenol. The strain was isolated from an acclimated mixed culture, and cultivated with phenols. They found that *Brevibacillus* sp. strain P-6 was able to completely remove phenol within 93 h of incubation, at concentrations lower than 200 mg L^–1^ (Yang and Lee, 2007). Overall, *Brevibacillus* genus has often been described as able to biodegrade organic contaminants, including contaminants with an aromatic ring, which could explain why it was highly more abundant in our soil after DZ and OXA introduction. This suggests that *Brevibacillus* strains were involved in the first biodegradation steps. This higher abundance of *Brevibacillus* is connected to the higher abundance of specific metabolic pathways (increased by more than 1,000% compared to native soil, Supplementary Table 4) highlighted with tax4fun tool. Some pathways involving Oxidoreductase, hydrolase, and lyase enzymes are acting on ester bonds, methane metabolism, or biosynthesis of secondary metabolites. These pathways were probably involved in the observed biotransformation of DZ and OXA, even if the high range of possible reactions involving these enzymes does not help to clearly identify the pathways.

*Hydrogenophaga* genus has often been described as able to biodegrade aromatic compounds such as polycyclic aromatic hydrocarbons (PAHs). A species from *Hydrogenophaga* genus was found to be relatively more abundant especially at T14 when most of DZ and OXA were degraded. Xin et al. (2010) showed that *Hydrogenophaga palleronii* LHJ38 grew on naphthalene as sole carbon and energy source, in minimal medium with 2 g L^–1^ naphthalene, highlighting its naphthalene biodegradation capacity (Xin et al., 2010). Yan et al. (2017) identified a PAHs-degrading strain, *Hydrogenophaga* sp. PYR1, isolated from PAH-contaminated river sediments. *Hydrogenophaga* sp. PYR1 was able to degrade high-molecular-weight polycyclic aromatic hydrocarbons (pyrene and Benzo[*a*]pyrene) under both aerobic and anaerobic conditions (Benzo[*a*]pyrene was degraded only under anaerobic conditions). These PAHs, being recalcitrant to microbial biodegradation, especially Benzo[*a*]pyrene, suggest that *Hydrogenophaga* may representa good candidate for bioremediation approaches (Yan et al., 2017). Yang et al. (2016) showed that *Hydrogenophaga atypical* QY7-2 efficiently degraded 3-Methyldiphenylether (MDE), an important alkyl-substituted diphenyl ether compound that is widely used as an intermediate in the synthesis of pyrethroid insecticides. The strain was isolated from activated sludge and was able to grow with MDE as sole carbon source, and to perform MDE complete mineralization, suggesting the ability to degrade aromatic rings (Yang et al., 2016). Gan et al. (2011) studied a co-culture consisting of *Hydrogenophaga* sp. PBC and *Ralstonia* sp. PBA, isolated from textile WWTP. The culture could tolerate and utilize 4-aminobenzenesulfonate (4-ABS) as sole carbon, nitrogen, and sulfur source under aerobic condition (Gan et al., 2011). *Hydrogenophaga* sp. H7 was also shown to simultaneously degrade 3-hydroxybenzoate (3-HBA) or 4-HBA (3-/4-HBA) during culture (Fan et al., 2019). Their results suggest that *Hydrogenophaga* sp. H7 is an ideal candidate for degradation of aromatic compounds. Regarding archaea, mainly Nitrosopumilaceae and Nitrososphaeraceae families presented specific evolutions, but in a lower extent compared to previously described bacteria, with a slightly higher abundance at T97. Both Nitrosopumilaceae and Nitrososphaeraceae belong to the Thaumarchaeota phylum, which is an important ammonia oxidizer group in different environments including soils, in aerobic conditions, and was the first archaeal phylum identified as involved in nitrification (Brochier-Armanet et al., 2012). Thaumarchaeota and especially Nitrosopumilaceae and Nitrososphaeraceae families are consequently chemolithoautotrophic organisms, able to grow *via* aerobic ammonia oxidation and CO_2_ fixation, with some organic material needed to support growth. The water from WWTP containing ammonia (approximately 0.2 mg/L), with regular inputs, could explain the presence of these taxa. The higher abundance observed at the end of our experiment could be linked to previous biodegradation of contaminants, leading to CO_2_ releases. All conditions could then have been reached during the experiment for the archaeal taxa growth. Thaumarchaeota may not have been specifically involved in DZ and OXA biodegradation, but their abundance could be part of the result of the biodegradation.

Regarding tax4Fun results, the highest impact is observed at T0, partly due to the *Brevibacillus* abundance at T0. These results concern only highly increased functions, but many others could have been necessary for benzodiazepine biodegradation. The tax4fun results may give perspectives for further studies, but it is not possible here to state on the different steps and enzymes involved in benzodiazepines biodegradation, as there are many, and it is not an obligation to observe an increase higher than 1,000% to explain a degradation pathway. For example, all function from group 3.5 (Acting on carbon-nitrogen bonds, other than peptide bonds), detected 131 times, increased between 15.7 and 28.3% compared to the native soil, with maximum at T14 after most of benzodiazepines were degraded. Such enzymes were probably necessary to perform benzodiazepine degradation, but their relative abundance did not increase significantly compared to the native soil. Functions increased by >1,000% were consequently probably involved in a high range of reactions and are not highly specific to benzodiazepine biodegradation (list of these functions in Supplementary Table 4). Most of these functions were detected mainly at T0. At T97, only two functions remain higher than 1,000% compared to the native soil, phenylalanine ammonia-lyase (EC:4.3.1.24) and NAD(P)H-quinone oxidoreductase subunit 6 (EC:1.6.5.3), suggesting that once biodegradation is completed, the soil returns to its original functions. If tax4fun provides a means to further exploit 16S rRNA genes dataset and draw hypotheses on the functional capabilities of microbial communities, results should be taken with caution as they remain only predictive and depend on functional information available from public genome databases.

Other functions decreased compared to the native soil (Table 2), but to a lower extent. Indeed, no function decreased lower than 100% compared to the native soil, and only 0.2–3.2% were decreased up to 50% compared to the native soil. This highlights that soil functions were conserved after benzodiazepines addition.

**TABLE 2:** Percentage of metabolic pathways (KEGG) increased or decreased compared to the native soil, at T0, T14, and T97.

		Percentage among the 3,554 metabolic pathways					
		T0D	T14D	T97D	T0O	T14O	T97O
Metabolic pathways increased compared to the native soil	>+10%	44.88	45.61	46.65	47.89	48.51	49.41
	>+50%	17.14	21.05	9.74	20.26	19.75	13.28
	>+100%	6.56	3.55	3.29	8.58	3.32	4.00
	>+500%	1.44	0.11	0.11	1.35	0.11	0.11
	>+1,000%	0.56	0.11	0.06	0.34	0.06	0.06
Metabolic pathways decreased compared to the native soil	<−10%	37.14	36.07	16.04	30.78	30.84	17.75
	<−50%	3.15	2.08	0.39	2.59	2.00	0.20
	<−100%	0.00	0.00	0.00	0.00	0.00	0.00

*For increased functions, results are presented for functions increased by 10, 50, 100, 500, and 1,000%; for decreased functions, results are presented for functions decreased by 10, 50, and 100%.*

### Fungal Candidates for Biodegradation of Diazepam and Oxazepam

Fungi are heterotrophic organisms. It is consequently harder to clearly link biodegradation of specific organic compounds to their abundance, especially when other carbon sources are available, which is the case in soils. It is, however, assumed that the abundance and growth of some specific OTUs in both microcosms (*n* = 3) suggest that these OTUs resisted DZ and OXA presence, and that they could have been involved in their biodegradation as their abundance increased over time. Six OTUs belonging to two phyla presented this evolution. Four OTUs belonged to Ascomycota and two OTUs belonged to Mortierellomycota (Figure 8B). Many *Mortierella* spp. are chitinolytic, and some species were described as able to degrade cellulose from plants to get sugar for their growth, *via* the xylanase enzymatic reaction. Ascomycota, as well as other fungi, require organic compounds as energy sources. This phylum has a wide range of metabolic capabilities and is also able to degrade biopolymers from plants such as lignin and cellulose (Boddy and Hiscox, 2017). It is admitted that fungi able to degrade complex biopolymers such as lignin or cellulose are also able to degrade recalcitrant organic compounds, such as polychlorobiphenyls (PCB), chlordecone, or polycyclic aromatic hydrocarbons (Cvancarova et al., 2012; Mao and Guan, 2016; Kadri et al., 2017). Consequently, the higher abundance of some OTUs belonging to Mortierellomycota and Ascomycota phyla after 97 days in our experiments could be linked to the biodegradation of the studied compounds.

Our results suggest that some specific OTUs evolved differently compared to others and were sometimes highly more abundant compared to the initial native soil. For bacteria and archaea, the shift in the community seemed to remain even after contaminant degradation, whereas for fungi, the structure of the community recovered values comparable to the native soil. A direct identification of degraders, bacteria, archaea, or fungi, is not possible in this kind of experiment, but it is interesting to observe the evolution and possible implications of specific OTUs, at the community level, as occurring in the environment. The biodegradation of the targeted compounds DZ and OXA when introduced onto soil was highly more rapid compared to the literature. This result suggests that the soil microbial community is well adapted to these CECs, due to regular inputs from WWTP, and that in that case, biodegradation can occur rapidly, with DT50 of less than 4 days in our study. The study site infiltration zone, being close from the sea and with low depth groundwater, is reassuring about the fate of DZ and OXA and the possible contamination of the sea. Moreover, the potential degradation/transformation by-products were not yet studied, and further studies will explore these aspects, to ensure that no more toxic and/or recalcitrant compounds are released by these biological reactions in soil.

## Conclusion

Biodegradation or biotransformation of DZ and OXA faster than previously described was observed in a soil receiving water from a WWPT, with a DT50 of less than 4 days. The analysis of the abundance, diversity, identity, and potential metabolisms of the soil bacterial, archaeal, and fungal communities has provided information on the identity of the microorganisms that potentially participated in benzodiazepines dissipation. The *Brevibacillus* and *Hydrogenophaga* bacterial genera especially presented specific evolution, with higher abundances right after DZ and OXA addition for *Brevibacillus*, and right after most DZ and OXA were degraded for *Hydrogenophaga*. These genera are known to degrade persistent organic contaminants, and seem to be good candidates to perform assays on benzodiazepine biodegradation. Future research on the metabolic capacities of *Brevibacillus* and *Hydrogenophaga* genera, as pure cultures or rather in bacterial, archaeal, and fungal communities in soil to promote cometabolism processes, could be explored. Moreover, the implication of the fungal Ascomycota, Mortierellomycota, or Rozellomycota phyla, which were found to be more abundant in the initial soil, on xenobiotics, and especially CEC biodegradation or biotransformation, could be explored. The abundance of Rozellomycota could represent a good bioindicator of a contamination by WWTP effluent rich in dissolved organic contaminants. A screening of this fungal OTU could be interesting in diverse contexts, especially in (ground)water impacted by WWTP effluents or sewage sludge spreading.

Even if disappearance of mother molecules DZ and OXA was clearly highlighted in the biotic soil microcosms in our study, it is so far not possible to clearly affirm if this was due to biodegradation (up to biodegradation by-products or to complete mineralization) or biotransformation of mother compounds. Performing a complete non-target screening of the different extracted samples, with the aim to identify the degradation compounds produced during the experiment and to confirm biodegradation or biotransformation pathways, is consequently planned.

## Data Availability Statement

All sequences were submitted to European Nucleotide Archive (ENA) database. 16S rRNA gene sequences and ITS2 sequences are available for the 19 samples under accession numbers ERS7290419 (SAMEA9567684) to ERS7290437 (SAMEA9567702). The whole project is referenced under accession number PRJEB47338.

## Author Contributions

MaC contributed to the experimental design, carried out experimental work, data analysis, and drafted the manuscript. CS, PS, JH, and CJ contributed to the experimental design, data analysis, and drafting and revising of the manuscript. GP-C, QG, and MP allowed access and provided information on the Agon-Coutainville site, and revised the manuscript. MiC carried out a part of the lab experiments in the study. All authors contributed to the article and approved the submitted version.

## Conflict of Interest

The authors declare that the research was conducted in the absence of any commercial or financial relationships that could be construed as a potential conflict of interest.

## Publisher’s Note

All claims expressed in this article are solely those of the authors and do not necessarily represent those of their affiliated organizations, or those of the publisher, the editors and the reviewers. Any product that may be evaluated in this article, or claim that may be made by its manufacturer, is not guaranteed or endorsed by the publisher.

## Footnotes

1
www.aquanes.eu

2
pbtprofiler.net
