# Supplementary material for: Dynamics of Soil Microbial Communities During Diazepam and Oxazepam Biodegradation in Soil Flooded by Water From a WWTP
Source: Front Microbiol. 2021 Nov 29;12:742000. doi: 10.3389/fmicb.2021.742000 (PMC8667618; doi:10.3389/fmicb.2021.742000)
Supplement: Supplementary file 1 [file Data_Sheet_1.docx]

Supplementary Material

**List of supplementary material**

[Supplementary Figure 1: Experimental setup used for biodegradation monitoring in diazepam and oxazepam spiked soil microcosms 2](#_Toc84346878)

[Supplementary Figure 2: Bacteria and Archaea abundance as 16S rRNA gene copies per g DW soil in (a) DZ microcosms and (b) OXA microcosms. Different letters correspond to significant differences (p<0.05, Kruskall Wallis test with Conover-Iman pair comparison). The – in each graph correspond to the values measured in the native initial soil. 2](#_Toc84346879)

[Supplementary Figure 3: Alpha diversity indices based on 16S rRNA clusters for bacteria and archaea on the different soil samples; (a) Number of observed OTUs, (b) Chao1 index, (c) Shannon index and (d) Inverse Simpson index. In green are the DZ conditions, in blue are the OXA conditions, without color filling is the native soil. Different letters correspond to significant differences (Kurskall-Wallis test with Conover-Iman comparison, P<0.05). 3](#_Toc84346880)

[Supplementary Figure 4: Fungal abundance as 18S rRNA gene copies per g DW soil in (a) DZ microcosms and (b) OXA microcosms. Different letters correspond to significant differences (p<0.05, Kruskall Wallis test with Conover-Iman pair comparison). The – in each graph correspond to the values measured in the native initial soil. 3](#_Toc84346881)

[Supplementary Figure 5: Alpha diversity indices based on fungal clusters for soil fungal community on the different soil samples; (a) Number of observed OTUs, (b) Chao1 index, (c) Shannon index and (d) Inverse Simpson index. Different letters correspond to significant differences (Kurskall-Wallis test with Conover-Iman comparison, P<0.05). 4](#_Toc84346882)

[Supplementary Table 1: Affiliation table for the 16S rRNA gene sequencing on the 19 samples, sequence processing and OTU affiliation using the Silva138_16S database 4](#_Toc84346883)

[Supplementary Table 2: Affiliation table for the ITS2 gene sequencing on the 19 samples, sequence processing and OTU affiliation using the Unite_Fungi_8.0_18112018 reference database 5](#_Toc84346884)

[Supplementary Table 3: Physico-chemical properties of the studied soil 6](#_Toc84346885)

[Supplementary Table 4: Metabolic pathways amplified by at least 1000% compared to the native soil. These results were obtained by using the Tax4Fun tool (Frogs, sygenae platform), based on 16S rRNA gene comparison with KEGG reference database 8](#_Toc84346886)


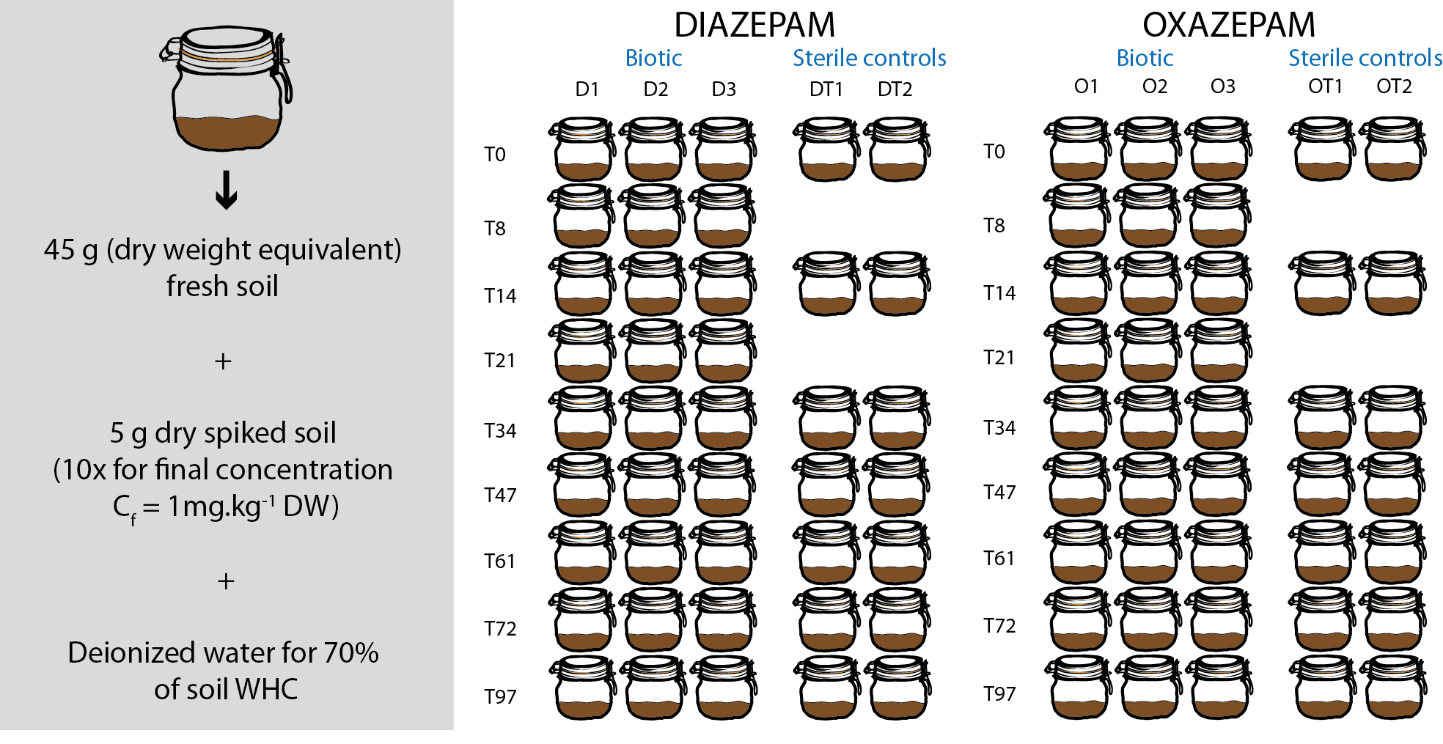


Supplementary Figure 1: Experimental setup used for biodegradation monitoring in diazepam and oxazepam spiked soil microcosms


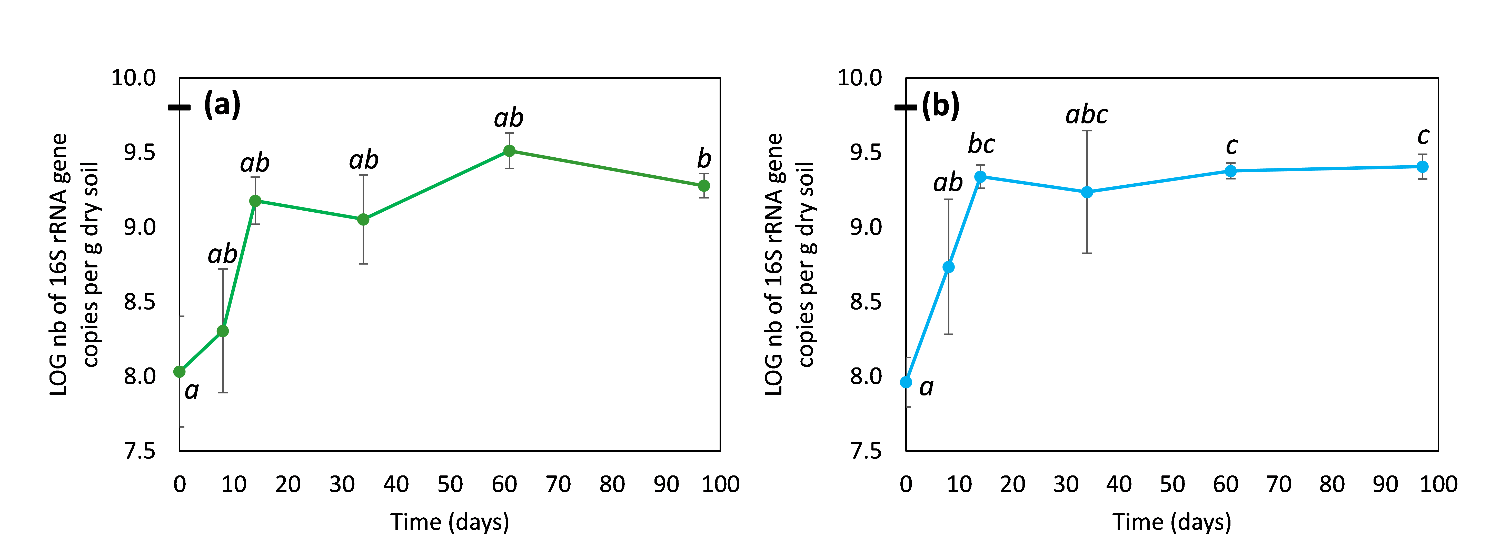


Supplementary Figure 2: Bacteria and Archaea abundance as 16S rRNA gene copies per g DW soil in (a) DZ microcosms and (b) OXA microcosms. Different letters correspond to significant differences (p<0.05, Kruskall Wallis test with Conover-Iman pair comparison). The – in each graph correspond to the values measured in the native initial soil.

Supplementary Figure 3: Alpha diversity indices based on 16S rRNA clusters for bacteria and archaea on the different soil samples; (a) Number of observed OTUs, (b) Chao1 index, (c) Shannon index and (d) Inverse Simpson index. In green are the DZ conditions, in blue are the OXA conditions, without color filling is the native soil.Different letters correspond to significant differences (Kurskall-Wallis test with Conover-Iman comparison, P<0.05).


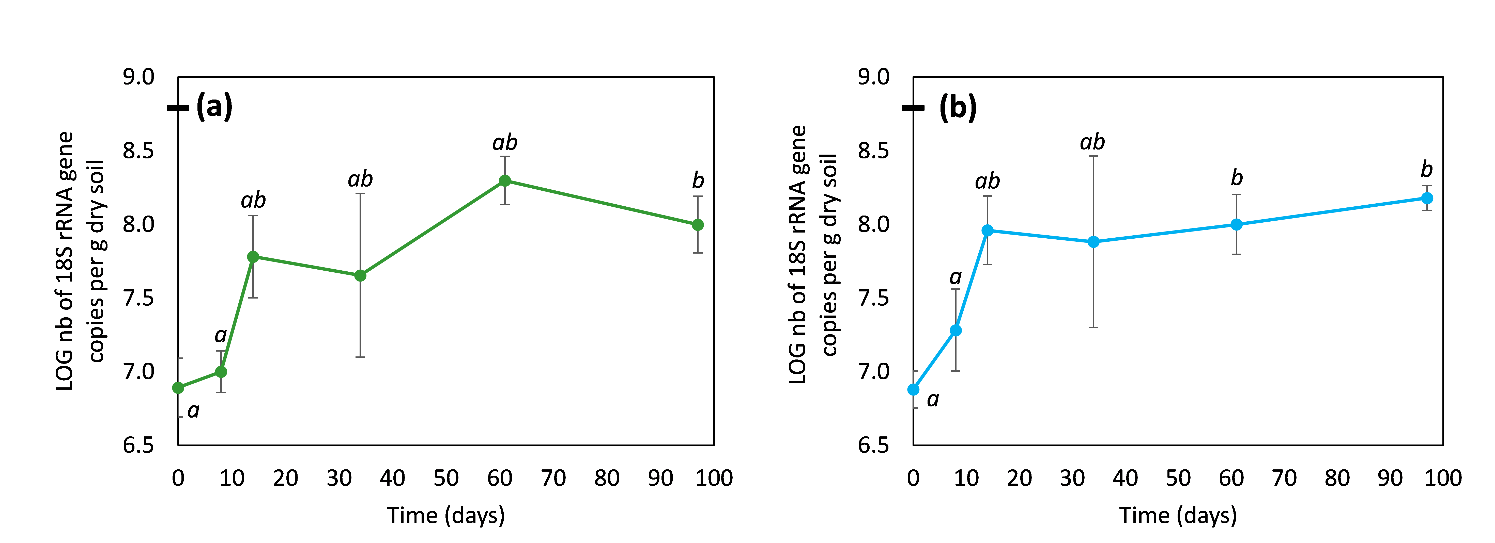


**Supplementary Figure 4: Fungal abundance as 18S rRNA gene copies per g DW soil in (a) DZ microcosms and (b) OXA microcosms. Different letters correspond to significant differences (p<0.05, Kruskall Wallis test with Conover-Iman pair comparison). The – in each graph correspond to the values measured in the native initial soil.**

Supplementary Figure 5: Alpha diversity indices based on fungal clusters for soil fungal community on the different soil samples; (a) Number of observed OTUs, (b) Chao1 index, (c) Shannon index and (d) Inverse Simpson index. Different letters correspond to significant differences (Kurskall-Wallis test with Conover-Iman comparison, P<0.05).

Supplementary Table 1: Affiliation table for the 16S rRNA gene sequencing on the 19 samples, sequence processing and OTU affiliation using the Silva138_16S database

|  | Sequences processing | | | Affilition stats | | | | | | |
| --- | --- | --- | --- | --- | --- | --- | --- | --- | --- | --- |
| Samples | Raw pre processed sequences with expected length | Kept sequences after trimming, chimera and artifacts removal | % kept for OTUs affiliation | Nb domain | Nb phylum | Nb class | Nb order | Nb family | Nb genus | Nb species |
| T0D1 | 35776 | 23033 | 64.38 | 2 | 36 | 93 | 209 | 318 | 480 | 668 |
| T0D2 | 36525 | 25056 | 68.60 | 2 | 36 | 93 | 206 | 321 | 489 | 677 |
| T0D3 | 30499 | 20415 | 66.94 | 2 | 35 | 91 | 204 | 316 | 481 | 663 |
| T14D1 | 35426 | 24321 | 68.65 | 2 | 34 | 90 | 202 | 314 | 478 | 655 |
| T14D2 | 34225 | 24434 | 71.39 | 2 | 36 | 93 | 210 | 325 | 495 | 689 |
| T14D3 | 37564 | 26835 | 71.44 | 2 | 35 | 93 | 212 | 325 | 495 | 691 |
| T97D1 | 29396 | 20714 | 70.47 | 2 | 35 | 92 | 207 | 320 | 488 | 672 |
| T97D2 | 32342 | 23154 | 71.59 | 2 | 36 | 94 | 212 | 324 | 492 | 675 |
| T97D3 | 33742 | 24197 | 71.71 | 2 | 35 | 93 | 213 | 324 | 491 | 678 |
| T001 | 30761 | 20109 | 65.37 | 2 | 35 | 92 | 207 | 318 | 482 | 671 |
| T002 | 31391 | 21050 | 67.06 | 2 | 35 | 93 | 206 | 318 | 482 | 668 |
| T003 | 32636 | 22597 | 69.24 | 2 | 35 | 91 | 207 | 321 | 491 | 678 |
| T1401 | 35196 | 24564 | 69.79 | 2 | 34 | 91 | 207 | 321 | 487 | 679 |
| T1402 | 33329 | 23092 | 69.29 | 2 | 36 | 94 | 211 | 323 | 480 | 669 |
| T1403 | 33498 | 23435 | 69.96 | 2 | 36 | 91 | 204 | 317 | 478 | 664 |
| T9701 | 34129 | 24430 | 71.58 | 2 | 36 | 92 | 212 | 323 | 494 | 684 |
| T9702 | 31636 | 22889 | 72.35 | 2 | 36 | 94 | 212 | 323 | 490 | 674 |
| T9703 | 32084 | 23009 | 71.71 | 2 | 36 | 94 | 211 | 324 | 493 | 678 |
| FR4N | 31820 | 22380 | 70.33 | 2 | 36 | 93 | 209 | 322 | 481 | 658 |

Supplementary Table 2: Affiliation table for the ITS2 gene sequencing on the 19 samples, sequence processing and OTU affiliation using the Unite_Fungi_8.0_18112018 reference database

|  | Sequence processing | | | Affiliation stats | | | | | | |
| --- | --- | --- | --- | --- | --- | --- | --- | --- | --- | --- |
|  | Raw pre-processed sequences with expected length | Kept sequences after trimming, chimera and artifacts removal | % kept for OTUs affiliation | Nb domain | Nb phylum | Nb class | Nb order | Nb family | Nb genus | Nb species |
| T0D1 | 44556 | 27110 | 60.8447796 | 1 | 10 | 25 | 49 | 92 | 125 | 148 |
| T0D2 | 41499 | 23483 | 56.5869057 | 1 | 11 | 26 | 48 | 96 | 127 | 145 |
| T0D3 | 40244 | 25249 | 62.7397873 | 1 | 11 | 25 | 47 | 100 | 126 | 143 |
| T14D1 | 38938 | 23472 | 60.2804458 | 1 | 9 | 24 | 46 | 101 | 143 | 171 |
| T14D2 | 39501 | 25021 | 63.3427002 | 1 | 12 | 27 | 51 | 105 | 143 | 174 |
| T14D3 | 36070 | 22308 | 61.8464098 | 1 | 12 | 26 | 47 | 102 | 139 | 169 |
| T97D1 | 42142 | 26031 | 61.7697309 | 1 | 12 | 27 | 50 | 104 | 142 | 175 |
| T97D2 | 36016 | 23123 | 64.2020213 | 1 | 11 | 25 | 47 | 99 | 134 | 159 |
| T97D3 | 40840 | 25868 | 63.3398629 | 1 | 12 | 26 | 46 | 97 | 137 | 164 |
| T001 | 9151 | 5141 | 56.1796525 | 1 | 9 | 22 | 40 | 84 | 112 | 131 |
| T002 | 41395 | 24247 | 58.5747071 | 1 | 10 | 25 | 46 | 96 | 125 | 144 |
| T003 | 41537 | 23568 | 56.7397742 | 1 | 12 | 26 | 46 | 92 | 122 | 142 |
| T1401 | 41021 | 24791 | 60.4348992 | 1 | 12 | 27 | 51 | 107 | 148 | 179 |
| T1402 | 35440 | 22019 | 62.1303612 | 1 | 13 | 28 | 51 | 103 | 141 | 166 |
| T1403 | 36982 | 23085 | 62.4222595 | 1 | 12 | 26 | 49 | 103 | 140 | 169 |
| T9701 | 30974 | 19878 | 64.176406 | 1 | 11 | 25 | 48 | 99 | 134 | 161 |
| T9702 | 37870 | 21232 | 56.0654872 | 1 | 11 | 25 | 47 | 99 | 137 | 168 |
| T9703 | 37651 | 21763 | 57.8019176 | 1 | 12 | 26 | 46 | 97 | 136 | 165 |
| FR4N | 37672 | 21893 | 58.1147802 | 1 | 12 | 27 | 52 | 110 | 150 | 183 |

Supplementary Table 3: Physico-chemical properties of the studied soil

|  |  | FR4N |
| --- | --- | --- |
| Soil type | | Calcareous sandy soil |
| Cation Exchange Capacity | CEC (meq/100g) | 8.5 |
|  | Ca / CEC (%) | 424.9 |
|  | K / CEC (%) | 3.4 |
|  | Mg / CEC (%) | 32.4 |
| Granulometry | Clays (%) | 2.6 |
|  | Fine silts (%) | 1.3 |
|  | coarse silts (%) | 5.8 |
|  | Fine sand (%) | 7.5 |
|  | Coarse sand (%) | 65.6 |
| C & N | OM (%) | 4.7 |
|  | Carbon (%) | 2.73 |
|  | N % | 0.24 |
|  | C/N | 11.5 |
| Geochemical parameters | pH water | 8.2 |
|  | pH KCl | 7.5 |
|  | CaCO3 Total (%) | 12.5 |
|  | CaO (mg/kg) | 10112 |
|  | P2O5 (mg/kg) | 65 |
|  | K2O (mg/kg) | 134 |
|  | MgO (mg/kg) | 550 |
| Metals / Metalloids | Cd (mg/kg DW) | 0.17 |
|  | Cr (mg/kg DW) | 9.9 |
|  | Cu (mg/kg DW) | 20.71 |
|  | Hg (mg/kg DW) | 0.06 |
|  | Ni (mg/kg DW) | 8.6 |
|  | Pb (mg/kg DW) | 7.08 |
|  | Zn (mg/kg DW) | 69.86 |

# Supplementary Table 4: Metabolic pathways amplified by at least 1000% compared to the native soil. These results were obtained by using the Tax4Fun tool (Frogs, sygenae platform), based on 16S rRNA gene comparison with KEGG reference database
